# Technology to Support Social, Health, and Well-Being Outcomes Among Older Adults

**DOI:** 10.1093/geroni/igab046.1645

**Published:** 2021-12-17

**Authors:** Walter Boot

## Abstract

In response to the COVID-19 pandemic, information and communication technologies (ICTs) are primarily how many people communicate, socialize, and receive healthcare. In a recent Pew report, experts in the role of technology in society believe that post-COVID-19 pandemic, society will continue to be far more technology-driven than pre-pandemic. That is, technology will play an even greater role in our lives in the “new normal.” However, compared to younger adults, many older adults are less likely to adopt the technologies needed to perform these everyday tasks. Differences in technology proficiency, acceptance, and adoption between groups is often referred to as the “digital divide,” and older adults are more likely to be on the disadvantaged side of this digital divide. This session explores the potential of technology to support social, health, and wellbeing outcomes among older adults, and the challenges involved. This session will start with a talk by A. Lothary on the success and challenges of using a simple video chat platform to address loneliness and social isolation. S. Shende will present a video-technology intervention for older adults with and without cognitive impairment, and how this intervention was designed to facilitate engagement. This will be followed by a presentation by X. Lin on the relationship between social media usage and well-being across the lifespan, and mediators of this relationship. The session will conclude with a presentation by W. Qin on predictors of older adults’ use of telehealth technology to support health and wellbeing during the COVID-19 pandemic.

